# Circulating Klotho and mortality patterns among US cancer survivors: A cohort study

**DOI:** 10.1097/MD.0000000000043471

**Published:** 2025-07-18

**Authors:** Jingying Nong, Yi Zhang

**Affiliations:** a Department of Thoracic Surgery, Xuanwu Hospital, Capital Medical University, Beijing, China.

**Keywords:** cancer, Klotho, mortality, NHANES

## Abstract

Klotho, a longevity hormone, exerts diverse anticancer activities. However, evidence regarding the association between serum Klotho and mortalities among cancer survivors is lacking. We examined the association between serum Klotho and the risks of all-cause and cancer mortalities among 1602 cancer adults from the National Health and Nutrition Examination Survey (NHANES) (2007–2016) using multivariate Cox proportional hazard models. The nonlinear relationship was determined using the likelihood ratios test, and the inflection points and 2-piecewise Cox proportional hazards regression models were computed. After a median follow-up period of 84.0 months, U-shaped associations between circulating Klotho and all-cause and cancer mortality were observed (*P* for nonlinear = .04, .02, respectively), with identified inflection points (pg/mL) of 765.5 for all-cause and 767.6 for cancer mortality. Klotho below these thresholds was inversely associated with all-cause mortality (Hazard ratio, HR, 95% confidence interval, CI) (0.72, 0.53–0.98) and cancer mortality (0.61, 0.39–0.96); Klotho above the threshold showed a trend of positive associated with cancer mortality (1.22, 0.99–1.50). Effect modification of age was apparent (*P* interaction .007); Klotho was associated positively with cancer mortality risk among participants aged under 60 (1.50, 1.09–2.05). The U-shaped associations between serum Klotho and all-cause and cancer mortality indicate that maintaining an ideal Klotho level in cancer patients could reduce mortality risks. This provides insight into the knowledge of nonlinearity relationship between serum Klotho, a longevity hormone, and survival outcomes in cancer populations.

## 1. Introduction

Cancer poses a threat to public health and a significant barrier to increasing life expectancy globally, with nearly 10 million cancer deaths for the year 2022.^[1]^ Investment in prognosis monitoring is one of the keys to reducing cancer deaths.

α-Klotho (Klotho), encoded by *KL*,^[2]^ was named after the Greek goddess who spun the thread of life and identified in 1997 by Kuro-o’s team as an aging suppressor.^[3]^ Its expression defect results in a spectrum of age-related disorders, whereas its overexpression extends life expectancy and health span by an impressive 30%.^[4]^ The discovery of Klotho in antiaging sparked immense interest and has led to a better understanding of its function in modulating signaling pathways and cardinal processes at cellular and organismal levels.^[5,6]^ Klotho plays a well-established role in regulating phosphate homeostasis and could attenuate inflammation and increase resistance to oxidative stress.^[7,8]^ There are 3 Klotho forms: a single-pass transmembrane protein, a secreted form, and shed Klotho, the proteolytic cleavage of the full-length transmembrane form.^[2]^ The tissue distribution of Klotho is mainly enhanced in the kidney, parathyroid gland, and placenta.^[3,9,10]^ Shed Klotho is primarily derived from the kidney and is found in sera, cerebrospinal fluid, and urine.^[11]^ Klotho deficiency has been linked to increased risks for a series of human aging-related disorders, including arteriosclerosis, diabetes mellitus, neurodegenerative conditions, chronic kidney diseases, or frailty in elderlies.^[6,12–14]^

Cancer and aging share similar molecular characteristics. Besides acting as a longevity hormone, Klotho could exert diverse anticancer activities by targeting many oncogenic pathways, including the insulin/IGF-1, Wnt, TGF-β1, mammalian target of rapamycin, and essential FGF pathways, some of which are essential for tumor survival and proliferation.^[5]^ Klotho deficiency participated in the development of various types of human malignancies. Klotho expression in cancer tissue was found to exhibit at a lower level than the adjacent tissues generally, inversely linked to histological grades and clinical stages, and served as a prognostic marker associated positively with survival in a spectrum of malignancies.^[8,15–17]^

Despite the ample literature on tissue Klotho in cancer, publications on serum Klotho and survival outcomes among cancer populations are rare. Serum Klotho was positively associated with survival in renal cell carcinoma in a retrospective single-center study.^[18]^ In general populations, some prospective studies investigated the association between circulating Klotho levels and cancer-related mortality and drew controversial conclusions.^[19,20]^

No study has prospectively assessed serum Klotho and survival outcomes in cancer populations. Against this background, we prospectively evaluated whether serum Klotho level is associated with all-cause and cancer mortality risks among cancer adults from the National Health and Nutrition Examination Survey (NHANES) 2007 to 2016.

## 2. Methods

### 2.1. Study design and population

- This prospective cohort study enrolled participants with cancer from 5 cycles of the 2-year NHANES dataset 2007 to 2016. NHANES was approved by the National Centre for Health Statistics Research Ethics Review Board. Participants provided written consent without being compensated or rewarded. The informed consent for using the public data is waived. The NHANES dataset includes a nationwide representative, noninstitutionalized US civilian sample with an intricate, multistage, stratified, clustered probability design. The survey combines interviews and physical examinations. The former occurs at respondents’ homes; health measurements are conducted in specially designed and equipped mobile examination centers. NHANES study procedures are described in more detail elsewhere (https://wwwn.cdc.gov/nchs/nhanes/analyticguidelines.aspx). The study reporting was guided by STROBE (Strengthening the Reporting of Observational Studies in Epidemiology).

**Figure 1.**
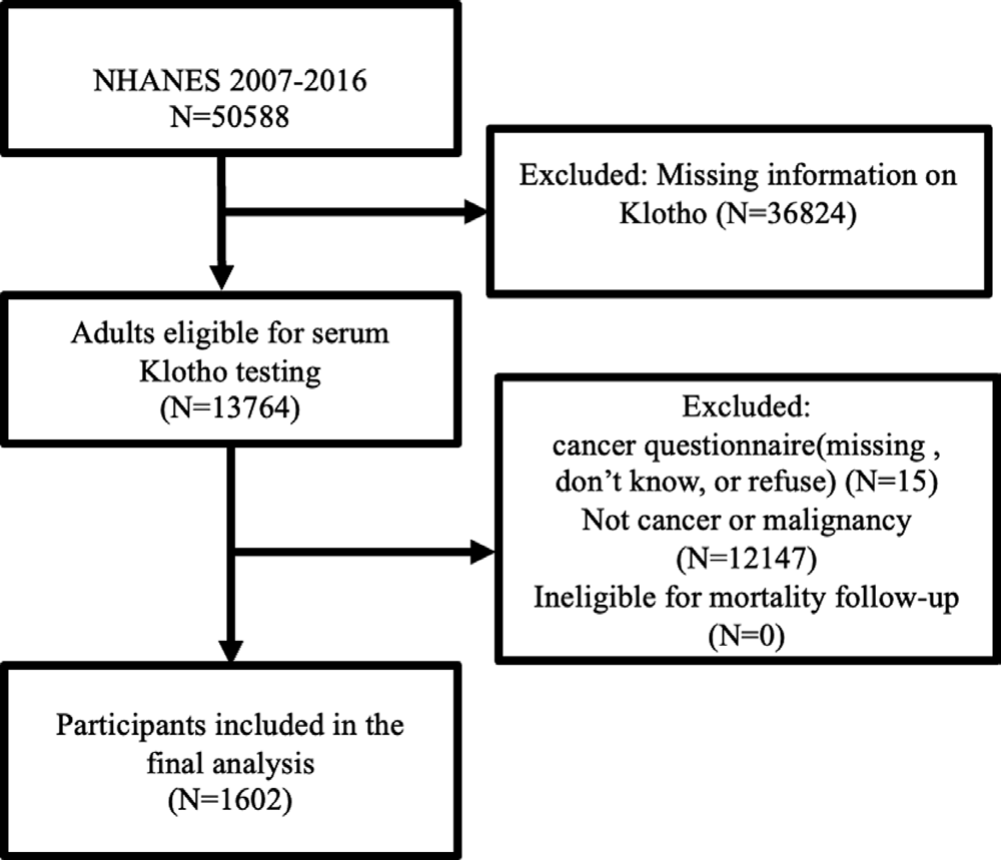
Screening flowchart of the study.

50,588 participants were included in the NHANES 2007 to 2016. Our study utilized existing NHANES data, and the detailed recruitment process pertains solely to the selection and preparation of the cancer cohort for analysis. We first excluded 36,824 participants missing information on Klotho, 13,764 adults eligible for the serum Klotho analysis were left. Responders who were missing or didn’t know/refused the cancer questionnaire (N = 15), denied cancers or malignancies (N = 12,147), or had no mortality follow-up information (N = 0) were excluded sequentially; 1602 cancer participants were enrolled in the final analyses (shown in Fig. 1).

### 2.2. Measurement of serum Klotho

All data of Klotho measurements were obtained from NHANES. Available pristine serum from responders aged 40 to 79 was collected. Samples were received under appropriate conditions, stored at −80°C, and analyzed with the ELISA method performed by a commercially available ELISA kit produced by IBL International, Japan.^[21]^ All sample analyses were duplicates, and the average was calculated as a final value. Remeasurements were flagged when the difference between duplicate values of a sample was over 10%. All results were checked for compliance with the laboratory’s standards before being released for reporting.

### 2.3. Cancer status

The NHANES provides a medical conditions section to report the interview data on self-reported health conditions. The diagnosis of cancer was based on a question modeled on this section: “Have you ever been told by a doctor or other health professional that you had cancer or a malignancy of any kind?”^[22]^ The question was asked at home employing the Computer-Assisted Personal Interview (CAPI) system.

### 2.4. Ascertainment of mortality

Mortality status, causes of death, and follow-up information were obtained from the linked mortality file (LMF) for public use by the National Death Index. All-cause mortality refers to death from any cause; cancer-related death (019–043) was according to the 10th version of the International Classification of Diseases (ICD-10). Mortality status was followed up through December 31, 2019.

### 2.5. Assessment of covariates

The demographic release file provides information on age, sex, race/ethnicity, and education level. Body mass index (BMI) was reported as weight in kilograms divided by height in square meters. In the personal interview data, coronary heart disease (CHD) and diabetes were determined according to the corresponding self-reported doctor-diagnosed questions. The one with chronic kidney disease awareness was defined by the question: “Ever told you had weak/failing kidneys, do not include kidney stones, bladder infections, or incontinence?”^[23]^ We defined a smoker as one who smoked no <100 cigarettes in their life, and an alcoholic drinker as having at least 12 alcoholic drinks per year.

### 2.6. Statistical analysis

Klotho was treated as a continuous variable scaled per 1-standard deviation (SD) increase or divided into quintiles, with the lowest Klotho quintile regarded as the reference. We reported continuous variables as survey-weighted mean (standard error, SE) and categorical variables as survey-weighted percentage and 95% confidence intervals (CIs). Multivariable Cox proportional hazards models computing hazard ratios (HRs) and 95% CIs were used to analyze the association of Klotho and mortalities. The follow-up duration was defined as the time from the baseline NHANES mobile examination center date until the date of death or the end of follow-up (December 31, 2019), whichever occurred first. Imputation of missing value was not conducted as missing data of any covariates was <5% (Table S1, Supplemental Digital Content, https://links.lww.com/MD/P488). When the analyses included the covariates that were missing, the missing participants were then omitted. Model 1 was adjusted for age and sex. Model 2 was additionally adjusted for race or ethnicity, education level, BMI, smoking history, alcohol drinking, CHD, diabetes, and chronic kidney disease awareness. Restricted cubic spline analysis with 3 knots (10th, 50th, and 90th percentiles) was used to explore the nonlinear association, and *P* nonlinear was computed using the likelihood ratios test. If a nonlinear association was observed, we computed the inflection point by a recursive algorithm and conducted 2-piecewise Cox proportional hazard models to estimate segments on both sides of the reflection point. Stratified analyses were performed by subgroups of sex, age (<60, ≥60), education level, BMI (<25, 25–30, ≥30), smoking, alcohol drinking, CHD, diabetes, and chronic kidney disease awareness by multivariate adjustment except for the stratification variable itself. A *P* value for the production terms was used to estimate the effect modification of stratified factors. In the sensitivity analyses, we repeated the multivariable Cox proportional hazards models for Klotho quintiles and mortalities, and the threshold effect analyses for the continuous Klotho and mortality risks with glomerular filtration rate (GFR) additionally adjusted based on Model 2; with GFR adjusted instead of chronic kidney disease awareness based on Model 2; or with time since cancer diagnosis additionally adjusted based on Model 2. The GFR was calculated to assess the glomerular function using the Cockcroft–Gault formula.^[24]^ A median value replaced the missing body weight value when calculating GFR. Time since cancer diagnosis was defined as the difference between the participant’s age at enrollment and their age at cancer diagnosis. All statistical analyses were conducted with R 4.3. *P *< .05 (2-tailed) was considered significant.

## 3. Results

### 3.1. Participant characteristics

The median follow-up time was 84.0 months (range 1.0–158.0 months). Table 1 shows the detailed demographics of the 1602 US cancer adults based on serum Klotho quintiles. Mean (SE) age, 62.8 (0.4) years; 765 male (45.9%) and 837 female (54.1%); Mexican American (2.3%), other Hispanic (2.1%), non-Hispanic White 1045 (86.7%), non-Hispanic Black 23 (4.7%), other race 90 (4.2%). Compared with the lowest Klotho quintile, cancer individuals with higher Klotho quintiles were younger, less likely to be smokers or alcoholic drinkers, less likely to have CHD or have chronic kidney disease awareness (*P* < .05).

**Table 1:** Characteristics of US adults with cancer in NHANES 2007–2016 based on the Klotho quintiles.

	Serum Klotho, pg/mL						*P* value
	Total	Quintile 1 (<594.8)	Quintile 2 (594.9–725.0)	Quintile 3 (725.1–842.5)	Quintile 4 (842.6–1011.9)	Quintile 5 (≥1012.0)	
Participants, N	1602	321	320	320	320	321	
Age (yr)	62.8 (0.4)	64.4 (0.6)	64.6 (0.7)	61.8 (0.8)	61.9 (0.8)	61.1 (0.7)	<.001
BMI	29.5 (0.2)	29.4 (0.4)	29.3 (0.4)	29.4 (0.5)	30.0 (0.5)	29.3 (0.4)	.787
Sex							
Male	765 (45.9)	166 (50.1)	177 (49.6)	155 (45.3)	136 (40.8)	131 (43.5)	.224
Female	837 (54.1)	155 (49.9)	143 (50.4)	165 (54.7)	184 (59.2)	190 (56.5)	
Race and ethnicity							
Mexican American	124 (2.3)	20 (1.8)	18 (1.3)	30 (2.9)	25 (2.5)	31 (3.2)	.014
Other Hispanic	112 (2.1)	20 (1.7)	15 (1.3)	17 (1.1)	32 (3.7)	28 (2.9)	
Non-Hispanic White	1045 (86.7)	216 (88.1)	224 (88.5)	219 (89.1)	209 (86.6)	177 (80.6)	
Non-Hispanic Black	231 (4.7)	49 (5.3)	41 (3.4)	38 (3.4)	36 (3.7)	67 (7.9)	
Other race	90 (4.2)	16 (3.2)	22 (5.6)	16 (3.5)	18 (3.6)	18 (5.4)	
Education level							
<High school	337 (10.9)	61 (10.7)	75 (11.7)	54 (9.7)	60 (10.0)	87 (12.7)	.280
High school graduate	340 (18.9)	78 (24.1)	63 (14.8)	72 (18.7)	69 (21.7)	58 (15.2)	
Some college or more	925 (70.1)	182 (65.3)	182 (73.4)	194 (71.6)	191 (68.3)	176 (72.1)	
Smoking							
yes	903 (55.8)	195 (61.2)	190 (58.6)	186 (59.0)	173 (53.8)	159 (45.0)	.023
No	698 (44.2)	125 (38.8)	130 (41.4)	134 (41.0)	147 (46.2)	162 (55.0)	
Drinking							
Yes	1104 (76.0)	246 (87.0)	223 (81.1)	225 (80.6)	214 (73.4)	196 (70.3)	.002
No	425 (20.7)	58 (13.0)	81 (18.9)	80 (19.4)	93 (26.6)	113 (29.7)	
Coronary heart disease							
Yes	125 (6.6)	45 (11.2)	25 (6.2)	16 (4.3)	17 (4.1)	22 (7.3)	.025
No	1469 (93.2)	274 (88.8)	294 (93.8)	300 (95.7)	302 (96.0)	299 (92.7)	
Diabetes							
Yes	338 (16.3)	79 (19.5)	58 (15.2)	70 (17.2)	62 (15.3)	69 (13.9)	.500
No	1263 (83.7)	241 (80.5)	262 (84.8)	250 (82.8)	258 (84.7)	252 (86.1)	
Chronic kidney disease awareness							
Yes	116 (5.9)	42 (9.9)	20 (6.7)	14 (3.4)	20 (4.8)	20 (4.9)	.063
No	1482 (94.0)	278 (90.1)	299 (93.3)	306 (96.6)	299 (95.2)	300 (95.2)	

Continuous variables: survey-weighted mean (SE); categorical variables: survey-weighted percentage (95% CI); continuous variables were compared using analysis of variance. Categorical variables were assessed with the Chi-square test.

BMI = body mass index, CI = confidence interval, NHANES = The National Health and Nutrition Examination Survey, SE = standard error.

**Figure 2.**
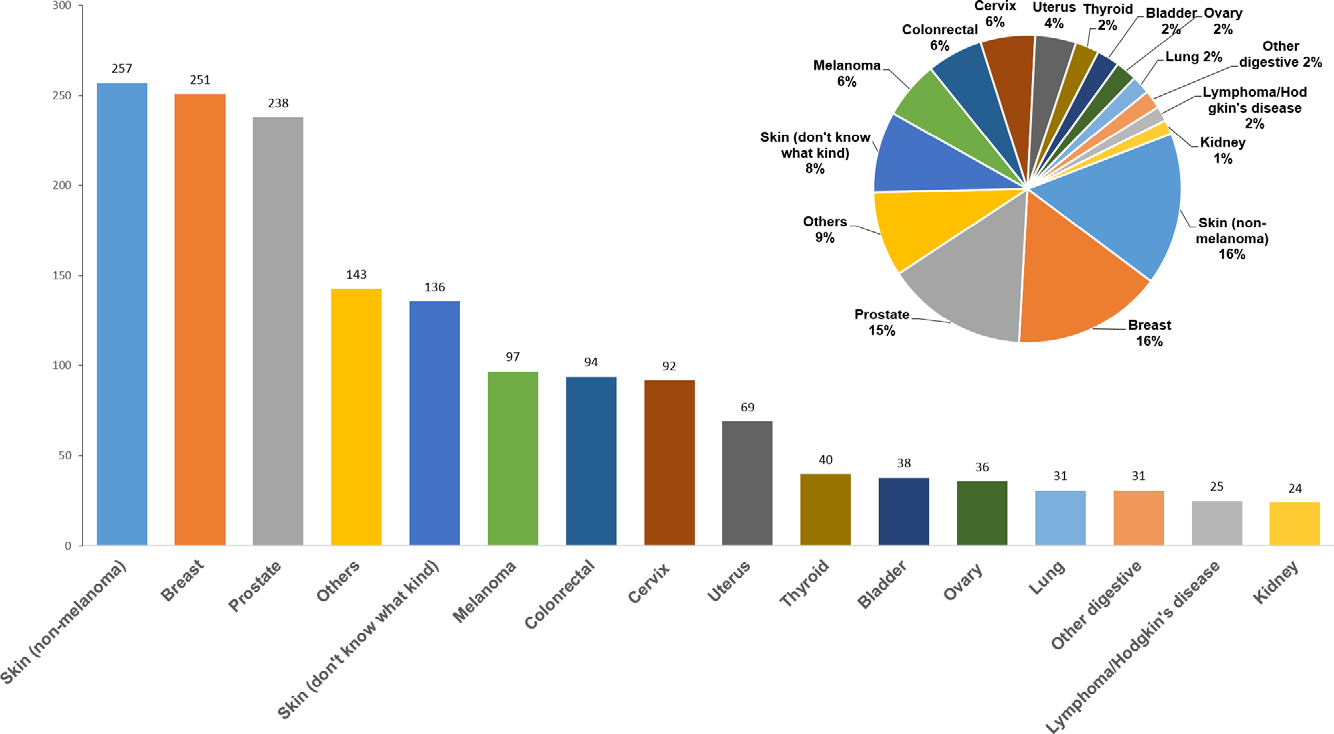
Distribution of cancer types among cancer survivors.

Statistical analyses of specific cancer types revealed 257 cases of non-melanoma skin cancer (16.0%), 251 cases of breast cancer (15.7%), 238 cases of prostate cancer (14.9%), 143 cases of cancer of others (8.9%) and 136 cases of skin cancer (don’t know what kind) (8.5%). These 5 cancer types represented the largest percentages in the study. Other digestive system cancers, including stomach, esophageal, pancreas, and liver, were grouped under “Other digestive” cancer due to the smaller sample sizes. Cancers with sample sizes smaller than 1%, as well as those reported as “Other,” were categorized under “Others” (Fig. 2).

### 3.2. Serum Klotho and mortalities

During the follow-up period, 341 all-cause deaths, with 147 deaths of cancer, were recorded. Cox proportional hazard models for all-cause mortality and cancer mortality based on serum Klotho quintiles are shown in Table 2. For all-cause mortality risk, compared with the reference group (quintile 1), the 3rd quintile (HR 0.66, 95% CI 0.48–0.92) and the 4th quintile (HR 0.69, 95% CI 0.49–0.96) of serum Klotho demonstrated lower all-cause mortality risks in Model 1; the 3rd quintile (HR 0.66, 95% CI 0.46–0.94) demonstrated lower all-cause mortality risk in Model 2. For cancer mortality, the 3rd quintile of serum Klotho showed a trend of lower cancer mortality risk in Model 2 (HR 0.63, 95% CI: 0.37–1.08, *P* = .09).

**Table 2:** Multivariable Cox regression for the associations between Klotho quintiles and mortalities in cancer participants.

Model	Hazard ratio (95% CI)				
	Quintile 1	Quintile 2	Quintile 3	Quintile 4	Quintile 5
All-cause mortality					
Deaths, No./total No.	82/321	70/320	62/320	58/320	69/321
Model 1	1 [ref]	0.75 (0.55, 1.03)	0.66 (0.48, 0.92)*	0.69 (0.49, 0.96)*	0.84 (0.61, 1.16)
Model 2	1 [ref]	0.74 (0.52, 1.05)	0.66 (0.46, 0.94)*	0.70 (0.49, 1.00)	0.80 (0.57, 1.13)
Cancer mortality					
Deaths, No./total No.	35/321	31/320	28/320	23/320	30/321
Model 1	1 [ref]	0.78 (0.48, 1.26)	0.70 (0.43, 1.16)	0.65 (0.38, 1.11)	0.87 (0.53, 1.41)
Model 2	1 [ref]	0.76 (0.45, 1.26)	0.63 (0.37, 1.08)	0.64 (0.36, 1.11)	0.86 (0.52, 1.43)

Model 1: Adjusted for age and sex; Model 2: Further adjusted for race/ethnicity, education level, body mass index (continuous), smoking, alcohol drinking, coronary heart disease, diabetes, and chronic kidney disease awareness.

CI = confidence interval.

* *P* < .05.

### 3.3. The detection of nonlinear associations

**Figure 3.**
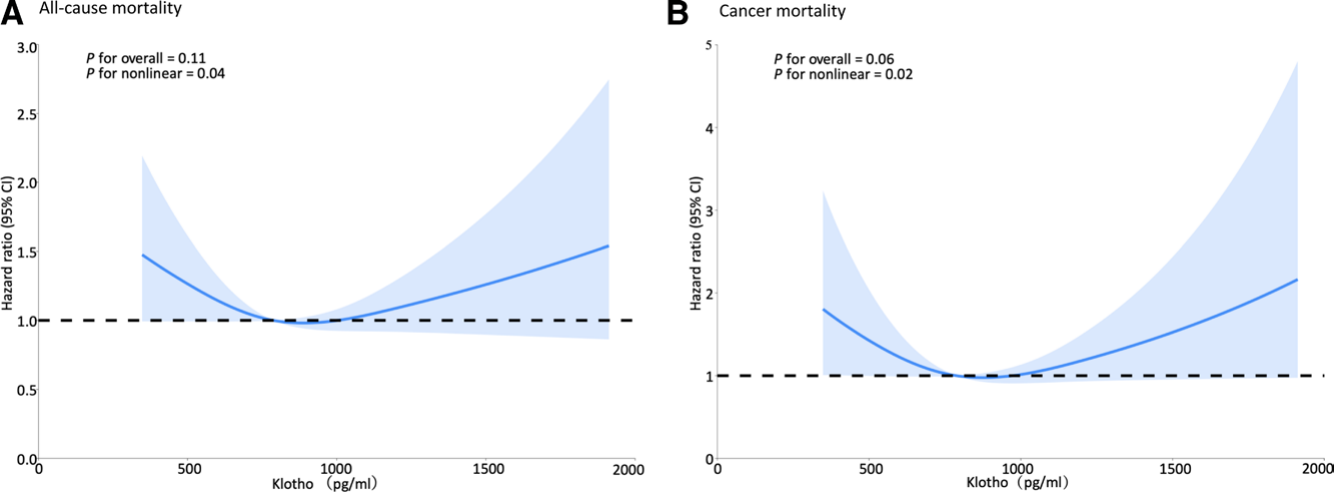
Restricted cubic splines show U-shape associations between serum Klotho and all-cause (A) and cancer mortality (B) in cancer survivors. The hazard ratio (solid line) was adjusted for age, sex, race, education level, body mass index, smoking history, alcohol drinking, coronary heart disease, diabetes, and chronic kidney disease awareness. Shaded areas represent 95% CIs. The model was conducted with 3 knots. CIs = confidence interval.

Restricted cubic splines with multiple adjustments showed U-shaped links between serum Klotho and all-cause (*P* nonlinear = .04) (Fig. 3A) and cancer mortality (*P* nonlinear = .02) (Fig. 3B). Threshold effect analyses computed inflection points of serum Klotho at 765.5 pg/mL for all-cause mortality and at 767.6 pg/mL for cancer mortality. Klotho below the threshold was linked inversely to all-cause mortality, with per 1-SD serum Klotho increase related to a 28% decrease in all-cause mortality risk (*P* = .039). Serum Klotho below the threshold was associated inversely with cancer mortality, per 1-SD increase of Klotho was associated with a 39% decrease in cancer mortality risk (*P* = .033); Klotho above the threshold showed a trend for positive association with cancer mortality, per 1-SD Klotho increase was associated with a 22% decrease in cancer mortality (*P* = .060) (Table 3).

**Table 3:** Multivariable threshold effect analysis for Klotho with all-cause and cancer mortality in cancer participants.

	Hazard ratio (95% CI)	*P* value
All-cause mortality		
Fitting by the standard Cox proportional risk model	1.00 (0.89, 1.12)	.926
Fitting by the 2-piecewise Cox proportional risk model		
Inflection point of Klotho (pg/mL)	765.5	
<765.5 pg/mL	0.72 (0.53, 0.98)	.039
≥767.6 pg/mL	1.12 (0.96, 1.29)	.147
*P* for log-likelihood ratio	.034	
Cancer mortality		
Fitting by the standard Cox proportional risk model	1.02 (0.86, 1.22)	.812
Fitting by the 2-piecewise Cox proportional risk model		
Inflection point of Klotho (pg/mL)	767.6	
<767.6 pg/mL	0.61 (0.39, 0.96)	.033
≥767.6 pg/mL	1.22 (0.99, 1.50)	.060
*P* for log-likelihood ratio	.021	

Adjusted for age (continuous), sex, race, education level, body mass index (continuous), smoking, alcohol drinking, coronary heart disease, diabetes, and chronic kidney disease awareness.

CI = confidence interval.

### 3.4. Stratified analyses

**Figure 4.**
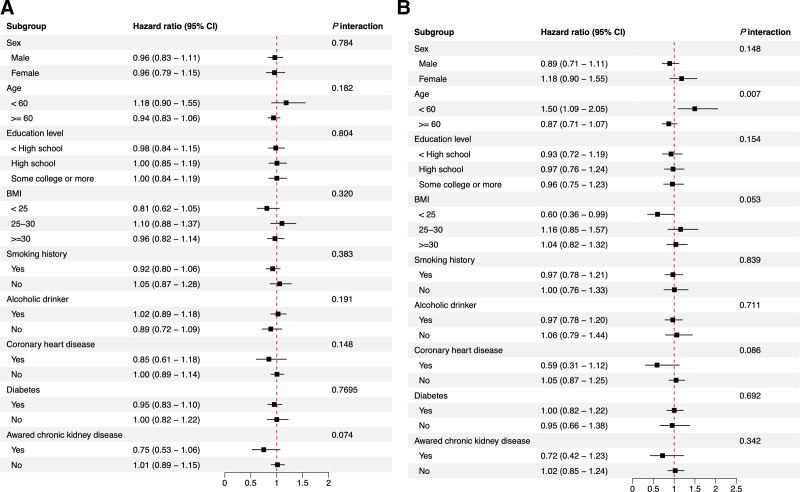
Cox regression for the associations between serum Klotho and all-cause (A) and cancer mortality (B) among subgroups. The analyses were adjusted for age (<60, ≥ 60), sex, education level, body mass index (<25, 25–30, ≥30), smoking, alcohol drinking, coronary heart disease, diabetes, and chronic kidney disease awareness. The strata variable was not included when stratification was performed on its own.

Effect modification by age was significant (*P* interaction = .007) for the association between Klotho and cancer mortality risk. Klotho was associated positively with cancer mortality risk among participants aged under 60 (1.50, 1.09–2.05). The effect modification of other strata variables on the association between serum Klotho and all-cause and cancer mortality was insignificant (Fig. 4A and B).

### 3.5. Sensitivity analyses

The results of the sensitivity analyses were generally consistent with the primary analyses when we repeated the Cox proportional hazard models and threshold effect analyses with GFR additionally adjusted based on Model 2 (Tables S2–S4, Supplemental Digital Content, https://links.lww.com/MD/P488); with GFR instead of chronic kidney disease awareness adjusted based on Model 2 (Tables S5 and S6, Supplemental Digital Content, https://links.lww.com/MD/P488); or with time since cancer diagnosis additionally adjusted based on Model 2 (Tables S7 and S8, Supplemental Digital Content, https://links.lww.com/MD/P488).

## 4. Discussion

In this prospective cohort study of 1602 cancer adults, a significant nonlinear association between serum Klotho and mortalities was observed, with identified threshold values of 765.5 pg/mL for all-cause mortality and 767.6 pg/mL for cancer mortality. Serum Klotho below these thresholds demonstrated an inverse association with all-cause mortality risk and cancer mortality risk; serum Klotho above these thresholds displayed a trend of positive association with the risk of cancer mortality. Age exhibited effect modification with Klotho for cancer mortality risk, serum Klotho was associated positively with cancer mortality among participants aged under 60. Sensitivity analyses did not find any profound changes, supporting the robustness of the primary findings.

Our study revealed a nonlinear U-shaped association between circulating Klotho and all-cause mortality. We did not find similar research in cancer patients. The circulating Klotho has been reported to be linked to all-cause mortality risks among the population with non-cancer illnesses such as chronic kidney disease,^[25,26]^ hypertension,^[27]^ severe abdominal aortic calcification^[28]^ and rheumatoid arthritis.^[29]^ The association between circulating Klotho and all-cause mortality risks has also been reported among general populations with controversial findings.^[14,19,20]^ A study included 804 community-dwelling participants aged ≥ 65 and found that individuals of the lowest Klotho tertile had higher mortality risk in the multivariate-adjusted Cox regression analyses.^[14]^ The other 2 studies of the NHANES database prospectively investigated the association of serum Klotho and all-cause mortality.^[19,20]^ Kresovich and Bulka^[19]^ reported that the group of the lowest Klotho exhibited an increased all-cause mortality risk than the group of the highest Klotho; an inverse link between continuous Klotho and all-cause mortality was observed, adjusted for age, sex and survey cycle. When alcohol consumption was further adjusted, the *P*-trend for categorized Klotho and the *P* value for the HR of continuous Klotho became insignificant. By contrast, Qiao et al^[20]^ did not observe a significant association between all-cause mortality with either Klotho tertiles or continuous Klotho. These inconsistent findings indicated a weak linear association between circulating Klotho and all-cause mortality. Regretfully, neither study explored nonlinear associations between Klotho and mortality. Consistent with our findings, a cohort study of 2608 frail participants with a median follow-up of 6.95 years also reported a U-shaped association between circulating Klotho levels and all-cause mortality risk in both crude and adjusted models^[30]^; however, that study did not specifically focus on cancer populations. Notably, our study provided preliminary evidence of such a U-shaped relationship between Klotho and all-cause mortality specifically among cancer survivors. Considering the diversity of cancer types included in our cohort and the possible heterogeneity in the association between Klotho and mortality across different cancer types, further validation in prospective studies focusing on specific cancer populations is warranted.

The current study found a U-shaped association between serum Klotho and cancer mortality, with serum Klotho below the inflection point demonstrating an inverse association and Klotho above the inflection point demonstrating a positive association. Two cohort studies in general populations have examined the association between circulating Klotho levels and cancer mortality, with both studies reporting nonsignificant findings. In a cohort study of 9879 participants with a follow-up period of 58 months, Qiao et al reported that there was no association between circulating Klotho levels and cancer-specific mortality, as determined by both the fine-gray test and multiple-adjusted Cox regression models.^[20]^ Similarly, Kresovich and Bulka^[19]^ found no significant association between circulating Klotho levels and cancer mortality in a cohort of 10,069 participants using multiple-adjusted Cox regression analysis. However, these 2 studies did not further explore the nonlinear relationship. A study of frail individuals reported a U-shaped relationship between serum Klotho levels and cancer-related mortality,^[30]^ demonstrating a similar pattern of association to that found in our study, albeit in a different population. These previous studies were predominantly composed of individuals without cancer, and the broader focus may dilute specific insights related to cancer patients. Klotho has anticancer activities. In the realm of cancer, more studies have been performed on tissue Klotho expression and illustrated its inverse association with cancer development and worsening prognoses^[5,17,31]^; publications on the association between serum Klotho levels and cancer-specific mortality risk are rare. Inconsistent with our results, a study of clear cell renal cell carcinoma found preoperative serum Klotho to be associated positively with cancer-specific and progression-free survival^[18]^; as a retrospective single-center study considering very limited covariates, the evidence was low quality. The antitumor properties of Klotho have made it a desirable therapeutic tool for cancer, and the idea is being discussed for elevating its levels in cancer patients locally or systemically.^[32,33]^ The U-shaped association demonstrated in our study here, as well as observed in frail non-cancer populations,^[30]^ underscores the importance of considering nonlinearity in biomarker-outcome relationships. It also suggests that maintaining a certain Klotho concentration might be more reasonable. Such an opinion that increased Klotho levels may not always improve health outcomes was supported by a few studies. An increase in Klotho activity was reported to cause a disorder characterized by hypophosphatemic rickets and hyperparathyroidism.^[34]^ Some research also indicated that the dosing or timing of Klotho delivery might tip the balance of benefits to harms and affect disorder processes.^[6,35,36]^ The reasons for such findings in cancer patients remain unclear; further elucidation by more studies from basic and clinical perspectives is needed.

Despite our estimation of the inflection points in the relationships between Klotho and mortality, it is imperative to acknowledge that the precise optimal level may vary in various populations due to different factors, such as demographic and clinical characteristics. Further prospective studies are necessary to validate these findings in diverse patient populations, which may facilitate the identification of more precise therapeutic targets of Klotho modulation in future clinical practice.

Notably, stratified analyses based on age demonstrated that elevated serum Klotho appeared to be harmful to cancer-specific survival outcomes in individuals aged under 60, indicating that Klotho might exert its effect in an age-specific manner. Similarly, a study reported that heterozygous individuals of the KL-VS (variant of *KL*) allele live longer beyond 70 but do not gain any advantage beyond 90 regardless of sex.^[37]^ Since Klotho-based therapeutics are becoming more likely to be translated as research advances, our findings provide clues for overcoming one of the critical challenges of precisely identifying who qualifies as a good candidate.

Our study has several strengths. Utilizing a prospective nationally representative sample, we provided preliminary evidence on the association between serum Klotho and all-cause and cancer mortality risks in cancer individuals. The analyses considered a multitude of detailed covariates as confounding factors. We explored the dose-response relationship between serum Klotho and mortality risks and identified nonlinear associations with inflection points. Although the sensitivity of the “weak or failing kidneys” question is imperfect in identifying all the responders with chronic kidney disease, GFR was adjusted additionally in the sensitivity analyses without profound changes observed. All these factors enhance the reliability of our findings.

Several limitations should be mentioned. First, we did not analyze the association among each subgroup of each cancer type since the present study included a broad spectrum of cancer types, resulting in modest numbers of participants and deaths for each specific cancer type, and the limited size for each cancer type significantly reduces the statistical power, undermining the reliability of subgroup analyses. Second, serum Klotho derived from a single specimen was less informative than serial testing. Third, residual confounding factors, such as the genotype, clinical-pathological characteristics and treatment of cancer participants, were absent and remain a concern. In addition, although we differentiated between all-cause mortality and cancer-specific mortality using ICD-10 codes, a further breakdown by specific cancer types or non-cancer causes of death was limited due to the small event numbers in each subgroup. A more granular analysis of cause-specific mortality requires future research with larger cohorts.

## 5. Conclusions

This prospective cohort study revealed U-shaped associations between serum Klotho level and all-cause and cancer mortalities among US cancer adults. Serum Klotho was associated positively with cancer mortality among participants aged under 60. Our findings suggest that maintaining an ideal Klotho level in cancer patients may be associated with reduced mortality risks. Our findings provide further insight into serum Klotho, a longevity hormone, and its nonlinear association with survival outcomes in cancer populations. More cohort studies of specific cancer types from basic and clinical perspectives are needed to elucidate the potential role of serum Klotho.

## Author contributions

**Conceptualization:** Jingying Nong, Yi Zhang.

**Data curation:** Jingying Nong.

**Formal analysis:** Jingying Nong.

**Funding acquisition:** Yi Zhang.

**Investigation:** Jingying Nong.

**Methodology:** Jingying Nong.

**Project administration:** Jingying Nong.

**Supervision:** Yi Zhang.

**Validation:** Jingying Nong.

**Visualization:** Jingying Nong.

**Writing – original draft:** Jingying Nong.

**Writing – review & editing:** Jingying Nong.

## Supplementary Material


