# Supplementary material for: Circulating Klotho and mortality patterns among US cancer survivors: A cohort study
Source: Medicine (Baltimore). 2025 Jul 18;104(29):e43471. doi: 10.1097/MD.0000000000043471 (PMC12282779; doi:10.1097/MD.0000000000043471)
Supplement: Supplementary file 1 [file medi-104-e43471-s001.docx]

eTable1. Missing data patterns

eTable 2 Characteristics of US adults with cancer in NHANES 2007-2016 based on the Klotho quintiles for sensitivity analyses

eTable 3 Multivariable Cox regression for the associations between Klotho quintiles and mortalities in cancer with GFR additionally adjusted based on model 2

eTable 4 Multivariable threshold effect analysis for Klotho with mortalities in cancer with GFR additionally adjusted based on model 2

eTable 5 Multivariable Cox regression for the associations between Klotho quintiles and mortalities in cancer with GFR instead of chronic kidney disease based on model 2

eTable 6 Multivariable threshold effect analysis for Klotho with mortalities in cancer with GFR instead of chronic kidney disease based on model 2

eTable 7 Multivariable Cox regression for the associations between Klotho quintiles and mortalities in cancer with time since cancer diagnosis additionally adjusted based on model 2

eTable 8 Multivariable threshold effect analysis for Klotho with mortalities in cancer with time since cancer diagnosis additionally adjusted based on model 2

eTable1. Missing data patterns

| Pattern | N | % |
| --- | --- | --- |
| Alcoholic drinker | 65 | 4.06 |
| Coronary heart disease | 6 | 0.37 |
| Awared chronic kidney disease | 3 | 0.19 |
| Diabetes | 1 | 0.06 |
| Smoking history | 1 | 0.06 |
| Body mass index | 16 | 1.00 |

eTable 2 Characteristics of US adults with cancer in NHANES 2007-2016 based on the Klotho quintiles for sensitivity analyses

| Characteristic | Participants, No. (weighted %) | | | | | | *P* value |
| --- | --- | --- | --- | --- | --- | --- | --- |
|  | Serum Klotho level, pg/ml | | | | | |  |
|  | Total | Quintile 1(<594.8) | Quintile 2 (594.9-725.0) | Quintile 3 (725.1-842.5) | Quintile 4 （842.6-1011.9） | Quintile 5 (≥1012.0) |  |
| **Participants**, No. | 1602 | 321 | 320 | 320 | 320 | 321 |  |
| **EGF** mean (SE) | 95.6(1.3) | 88.2(2.6) | 90.2(2.1) | 97.1(2.9) | 102.4(2.9) | 100.2(2.7) | < 0.001 |
| **Time since diagnosis mean** (SE) (year) | 10.8(0.4) | 10.8(0.8) | 10.5(0.8) | 11.3(0.8) | 10.4(0.7) | 11.1(0.9) | 0.893 |
| Continuous variables: survey-weighted mean (SE)  Categorical variables: survey-weighted percentage (95% CI)  Continuous variables were compared using analysis of variance. Categorical variables were assessed with the Chi-square test.  SE, standard error; EGF, estimated glomerular filtration rate; CI, confidence interval | | | | | | | |

| eTable 3 Multivariable Cox regression for the associations between Klotho quintiles and mortalities in cancer with GFR additionally adjusted based on model 2 | | | | | |
| --- | --- | --- | --- | --- | --- |
| Model | Hazard ratio (95% CI) | | | | |
|  | Quintile | Quintile 2 | Quintile 3 | Quintile 4 | Quintile 5 |
| **All-cause mortality** | | | | | |
| Deaths, No./total No. | 82/321 | 70/320 | 62/320 | 58/320 | 69/321 |
| Model | 1[ref] | 0.74 (0.52, 1.04) | 0.66 (0.46, 0.94) * | 0.72 (0.50, 1.04) | 0.81 (0.58, 1.15) |
| **Cancer mortality** | | | | | |
| Deaths, No./total No. | 35/321 | 31/320 | 28/320 | 23/320 | 30/321 |
| Model | 1[ref] | 0.74 (0.44, 1.23) | 0.62 (0.36, 1.07) | 0.63 (0.36, 1.10) | 0.84 (0.50, 1.40) |

**P* < 0.05

Model: Adjusted for age (continuous), sex, race, education level, body mass index (continuous), smoking history, alcohol drinking, coronary heart disease, diabetes, chronic kidney disease awareness, and GFR

GFR, Estimated glomerular filtration rate

| eTable 4 Multivariable threshold effect analysis for Klotho with mortalities in cancer with GFR additionally adjusted based on model 2 | | |
| --- | --- | --- |
|  | Hazard ratio (95% CI) | *P* value |
| **All-cause mortality** |  |  |
| Fitting by the standard Cox proportional risk model | 1.01 (0.90, 1.13) | 0.923 |
| Fitting by the two-piecewise Cox proportional risk model | |  |
| Inflection point of Klotho (pg/ml) | 765.0 |  |
| < 765.0 pg/ml | 0.74 (0.54, 1.01) | 0.058 |
| ≥765.0 pg/ml | 1.12 (0.97, 1.30) | 0.131 |
| *P* for Log-likelihood ratio | 0.043 |  |
| **Cancer mortality** |  |  |
| Fitting by the standard Cox proportional risk model | 1.02 (0.86, 1.21) | 0.829 |
| Fitting by the two-piecewise Cox proportional risk model | |  |
| Inflection point of Klotho (pg/ml) | 767.6 |  |
| < 767.6 pg/ml | 0.61 (0.39, 0.95) | 0.030 |
| ≥767.6 pg/ml | 1.22 (0.99, 1.50) | 0.062 |
| *P* for Log-likelihood ratio | 0.020 |  |

Adjusted for age, sex, education level, body mass index, smoking history, alcohol drinking, chronic heart disease, diabetes, chronic kidney disease awareness, and GFR_._

GFR, Estimated glomerular filtration rate

| eTable 5 Multivariable Cox regression for the associations between Klotho quintiles and mortalities in cancer with GFR instead of chronic kidney disease based on model 2 | | | | | |
| --- | --- | --- | --- | --- | --- |
| Model | Hazard ratio (95% CI) | | | | |
|  | Serum Klotho level, pg/ml | | | | |
|  | Quintile 1 | Quintile 2 | Quintile 3 | Quintile 4 | Quintile 5 |
| **All-cause mortality** | |  |  |  |  |
| Deaths, No./total No. | 82/321 | 70/320 | 62/320 | 58/320 | 69/321 |
| Model | 1[ref] | 0.70 (0.50, 0.99) * | 0.64 (0.45, 0.91) * | 0.73 (0.51, 1.04) | 0.80 (0.56, 1.12) |
| **Cancer mortality** | |  |  |  |  |
| Deaths, No./total No. | 35/321 | 31/320 | 28/320 | 23/320 | 30/321 |
| Model | 1[ref] | 0.70 (0.42, 1.16) | 0.59 (0.35, 1.02) | 0.64 (0.37, 1.11) | 0.81 (0.49, 1.35) |

**P* < 0.05

Model: adjusted for age (continuous), sex, race, education level, body mass index (continuous), smoking history, alcohol drinking, coronary heart disease, diabetes, and GFR

GFR: Estimated glomerular filtration rate

| eTable 6 Multivariable threshold effect analysis for Klotho with mortalities in cancer with GFR instead of chronic kidney disease based on model 2 | | |
| --- | --- | --- |
|  | HR (95% CI) | *P* value |
| **All-cause mortality** |  |  |
| Fitting by the standard Cox proportional risk model | 1.01 (0.90, 1.13) | 0.903 |
| Fitting by the two-piecewise Cox proportional risk model | |  |
| Inflection point of Klotho (pg/ml) | 764.3 |  |
| < 764.3 pg/ml | 0.72 (0.53, 0.98) | 0.035 |
| ≥764.3 pg/ml | 1.14 (0.98, 1.32) | 0.088 |
| *P* for Log-likelihood ratio | 0.024 |  |
| **Cancer mortality** |  |  |
| Fitting by the standard Cox proportional risk model | 1.02 (0.86, 1.22) | 0.816 |
| Fitting by the two-piecewise Cox proportional risk model | |  |
| Inflection point of Klotho (pg/ml) | 762.9 |  |
| < 762.9 pg/ml | 0.58 (0.37, 0.92) | 0.019 |
| ≥762.9 pg/ml | 1.24 (1.01, 1.52) | 0.044 |
| *P* for Log-likelihood ratio | 0.013 |  |

Adjusted for age, sex, race, education level, body mass index, smoking history, alcohol drinking, chronic heart disease, diabetes, and GFR.

GFR, Estimated glomerular filtration rate

| eTable 7 Multivariable Cox regression for the associations between Klotho quintiles and mortalities in cancer with time since cancer diagnosis additionally adjusted based on model 2 | | | | | |
| --- | --- | --- | --- | --- | --- |
| Model | Hazard ratio (95% CI) | | | | |
|  | Quintile 1 | Quintile 2 | Quintile 3 | Quintile 4 | Quintile 5 |
| **All-cause mortality** | |  |  |  |  |
| Deaths, No./total No. | 82/321 | 70/320 | 62/320 | 58/320 | 69/321 |
| Model | 1[ref] | 0.70 (0.49, 0.99) * | 0.63 (0.44, 0.90) * | 0.69 (0.48, 0.99) * | 0.77 (0.54, 1.08) |
| **Cancer mortality** | |  |  |  |  |
| Deaths, No./total No. | 35/321 | 31/320 | 28/320 | 23/320 | 30/321 |
| Model | 1[ref] | 0.70 (0.42, 1.18) | 0.60 (0.35, 1.05) | 0.59 (0.33, 1.04) | 0.79 (0.47, 1.34) |

**P* < 0.05

Adjusted for age, sex, race, education level, body mass index (continuous), smoking history, alcohol drinking, coronary heart disease, diabetes, chronic kidney disease awareness, and time since cancer diagnosis

| eTable 8 Multivariable threshold effect analysis for Klotho with mortalities in cancer with time since cancer diagnosis additionally adjusted based on model 2 | | |
| --- | --- | --- |
|  | Hazard ratio (95% CI) | *P* value |
| **All-cause mortality** |  |  |
| Fitting by the standard Cox proportional risk model | 0.99 (0.88, 1.11) | 0.858 |
| Fitting by the two-piecewise Cox proportional risk model | |  |
| Inflection point of Klotho (pg/ml) | 765.0 |  |
| < 765.0 pg/ml | 0.71 (0.52, 0.96) | 0.028 |
| ≥765.0 pg/ml | 1.12 (0.96, 1.29) | 0.144 |
| *P* for Log-likelihood ratio | 0.026 |  |
| **Cancer mortality** |  |  |
| Fitting by the standard Cox proportional risk model | 1.01 (0.85, 1.21) | 0.897 |
| Fitting by the two-piecewise Cox proportional risk model | |  |
| Inflection point of Klotho (pg/ml) | 752.2 |  |
| < 752.2 pg/ml | 0.57 (0.36, 0.92) | 0.022 |
| ≥752.2 pg/ml | 1.21 (0.99, 1.50) | 0.068 |
| *P* for Log-likelihood ratio | 0.017 |  |

Adjusted for age, sex, education level, body mass index, smoking history, alcohol drinking, coronary heart disease, diabetes, chronic kidney disease awareness, and time since cancer diagnosis.
